# Loss of *ALN* Function in *Arabidopsis thaliana* Induces Allantoin Accumulation and Enhances Antioxidant Enzyme Responses to Copper Toxicity

**DOI:** 10.3390/plants15193074

**Published:** 2026-10-08

**Authors:** Klaudia Bylińska, Beata Michniewska, Weronika Rusin, Moshe Sagi, Edyta Zdunek-Zastocka

**Affiliations:** 1 Department of Biochemistry and Microbiology, Warsaw University of Life Sciences-SGGW, Nowoursynowska 159, 02-776 Warsaw, Poland; klaudia_bylinska@sggw.edu.pl (K.B.); beata_michniewska@sggw.edu.pl (B.M.); weronika_kusek@sggw.edu.pl (W.R.); 2 The Albert Katz Department of Dryland Biotechnologies, French Associates Institute for Agriculture and Biotechnology of Dryland, The Jacob Blaustein Institutes for Desert Research, Ben-Gurion University of the Negev, Sde Boker Campus, Midreshet Ben-Gurion 8499000, Israel; gizi@bgu.ac.il

**Keywords:** copper toxicity, ureide metabolism, allantoinase, *Arabidopsis thaliana*, oxidative stress, antioxidant enzymes

## Abstract

Environmental contamination with heavy metals severely impairs crop productivity, primarily by inhibiting growth and triggering oxidative damage. To counteract these adverse effects, plants undergo complex metabolic reprogramming. Among these adaptations, the accumulation of purine-derived ureides—particularly allantoin—has been proposed as a protective mechanism, though its role under copper (Cu) stress remains unexplored. Here, we demonstrate that wild-type (*Arabidopsis thaliana*, WT) plants accumulate allantoate—the product of allantoin hydrolysis catalyzed by allantoinase (ALN, EC 3.5.2.5)—rather than allantoin when exposed to Cu toxicity. However, blocking this downstream catabolic step via loss of *ALN* function results in allantoin accumulation and is associated with enhanced Cu tolerance. Under Cu stress, *aln-1* and *aln-4* loss-of-function mutants maintained higher biomass and accumulated lower levels of malondialdehyde, superoxide anions, and hydrogen peroxide compared to WT. Furthermore, *aln* lines exhibited a stronger Cu-induced activation of key antioxidant enzymes (SOD, CAT, POD, APX) alongside higher expression of genes encoding specific isoforms targeted to multiple cellular compartments: cytosolic (*CSD1*), chloroplastic (*CSD2*, *tAPX*), peroxisomal (*APX3*, *CAT3*), and apoplastic (*Prx51*, *Prx47*). These results reveal that the loss of *ALN* function promotes a multi-compartmental antioxidant response under Cu toxicity, highlighting the pivotal role of purine catabolism in plant redox homeostasis and pointing to ureide metabolism as a potential avenue for enhancing crop stress tolerance.

## 1. Introduction

The increasing accumulation of copper (Cu) in agricultural soils poses a significant environmental challenge, adversely affecting plant growth and productivity. Elevated Cu concentrations may result from mining, industrial emissions, wastewater irrigation, and the repeated use of Cu-based agrochemicals, leading to its accumulation in soils and uptake by plants [1,2]. Although Cu is required in trace amounts for essential cellular processes, including photosynthetic and respiratory electron transport and the activity of numerous metalloenzymes, excessive Cu availability disrupts physiological and biochemical processes. High Cu concentrations can impair root development, nutrient acquisition, photosynthesis, and carbon and nitrogen metabolism, ultimately restricting plant growth and biomass production [3,4,5].

A major component of Cu phytotoxicity is oxidative stress. Excess Cu promotes the generation of reactive oxygen species (ROS) through Cu-mediated redox reactions and impairment of electron transport processes in chloroplasts and mitochondria [6]. The resulting accumulation of superoxide anions (O_2_^−^) and hydrogen peroxide (H_2_O_2_) can cause lipid peroxidation, protein oxidation, nucleic acid damage, and membrane deterioration [7,8]. Plants counteract these effects through an integrated antioxidant network comprising enzymatic and non-enzymatic components. Superoxide dismutases (SODs) convert O_2_^−^ into H_2_O_2_, which is subsequently detoxified by catalases (CATs), ascorbate peroxidases (APXs), and class III peroxidases (PODs), together with low-molecular-weight antioxidants such as ascorbate, glutathione, and proline [9,10]. The coordinated regulation of these defense systems across different cellular compartments is therefore crucial for adaptation to Cu-induced oxidative stress.

Metabolic reprogramming is a fundamental component of plant adaptation to abiotic stress. Among the metabolic pathways increasingly associated with stress responses is purine catabolism, which generates the nitrogen-rich ureides—allantoin and allantoate [11,12]. In tropical legumes such as soybean (*Glycine max*) and common bean (*Phaseolus vulgaris*), ureides serve as major long-distance nitrogen transport compounds, facilitating the export of fixed nitrogen from root nodules to shoot tissues [13,14]. In non-leguminous species, like *Arabidopsis thaliana*, ureides contribute to nitrogen recycling and remobilization and are involved in metabolic adaptation under nitrogen and carbon deprivation [15,16].

Among ureides, allantoin has attracted increasing attention due to its marked accumulation under a broad range of environmental conditions, including drought, osmotic and salt stress, ammonium excess, prolonged darkness, high irradiance, wounding, UV-C irradiation, sulfate deficiency, and cadmium (Cd) stress [17,18,19,20,21,22]. Furthermore, both genetic disruption of allantoin catabolism and exogenous application of allantoin have been shown to enhance plant tolerance to multiple abiotic stresses—such as salinity, drought, and heat, as well as arsenic and Cd toxicity [22,23,24,25,26,27,28]. These findings point to a broader role for allantoin in stress adaptation, extending beyond its established functions in purine catabolism and nitrogen recycling. However, ureide accumulation patterns may depend on the type of stress and plant species. For example, drought stress in common beans predominantly increases allantoate rather than allantoin levels [29]. In contrast, Cd stress elevates both ureides in this species, whereas in pea (*Pisum sativum*), it reduces their levels in leaves while increasing them in roots [22].

Ureides are intermediate products of purine catabolism generated through enzymatic reactions occurring across several cellular compartments [30,31]. Following the conversion of different purine substrates to xanthine, xanthine dehydrogenase (XDH) catalyzes the first common step in ureide biosynthesis, converting xanthine to uric acid in the cytosol [18,32]. Uric acid is subsequently transported to the peroxisome, where it is converted to allantoin. Allantoin is then transported to the endoplasmic reticulum, where it is hydrolyzed to allantoate by allantoinase (ALN; EC 3.5.2.5) [33]. Further ureide degradation releases nitrogen and carbon skeletons that can be reassimilated into primary metabolism [34]. Thus, ALN may represent a key regulatory step in allantoin turnover and downstream ureide catabolism, with the potential to influence both allantoin accumulation and nitrogen recycling.

In Arabidopsis, loss of *ALN* function leads to increased allantoin accumulation and enhanced tolerance to drought and polyethylene glycol (PEG)-induced osmotic stress [35]. This response has been associated with stimulation of abscisic acid (ABA) metabolism, including increased expression of *NCED3*, which encodes 9-cis-epoxycartenoid dioxygenase 3, a key enzyme of ABA biosynthesis, and post-translational activation of the β-glucosidase BG1, which releases active ABA from its conjugated form [35]. The *aln-1* and *aln-2* mutants also exhibit broad transcriptional reprogramming involving stress-responsive genes, including those associated with ABA signaling and abiotic stress responses [35]. In addition, increased allantoin accumulation in the *aln-3* mutant has been associated with reduced ROS accumulation and enhanced activities of antioxidant enzymes, including SOD and APX, under Cd stress [36]. Together, these findings suggest that altered ureide metabolism, particularly increased allantoin availability, may contribute to stress tolerance through interconnected mechanisms involving ABA signaling, transcriptional regulation, and redox homeostasis.

Although allantoin accumulation has been associated with plant responses to several abiotic stresses, the effect of excess Cu on ureide pools remains unexplored. Whether and to what extent Cu stress reconfigures the ureide profile, and how loss of *ALN* function affects plant tolerance to Cu toxicity, remain to be elucidated. To address these questions, we characterized WT and two independent *ALN* loss-of-function mutants, *aln-1* and *aln-4*, under Cu stress. We analyzed allantoin and allantoate levels, plant growth, oxidative damage, ROS accumulation, antioxidant enzyme activities, and the expression of genes encoding antioxidant isoforms targeted to distinct subcellular compartments. By integrating physiological, biochemical, and molecular approaches, we aimed to determine whether *ALN* deficiency and the associated changes in ureide metabolism contribute to plant responses to Cu stress and whether these changes are linked to the regulation of antioxidant defenses across multiple cellular compartments.

## 2. Results

### 2.1. Impact of Cu Stress on Ureide Accumulation

**Figure 1:**
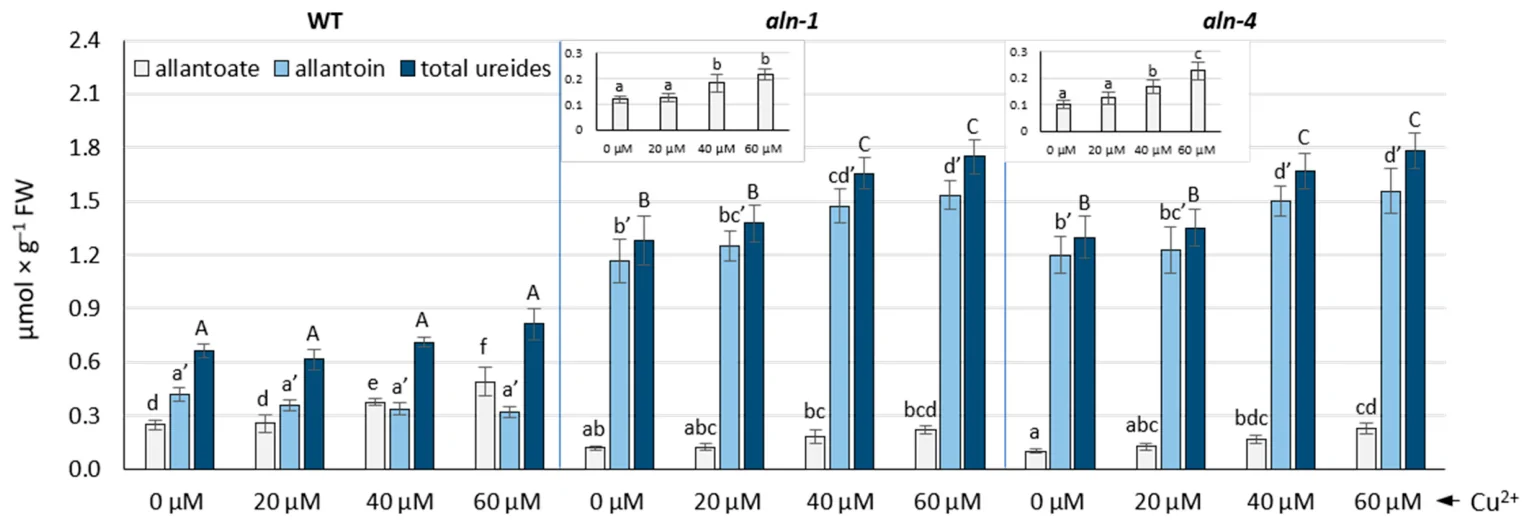
Effect of copper exposure on the levels of allantoate, allantoin, and total ureides in 23-day-old wild-type (WT) plants and mutant lines (*aln-1*, *aln-4*). Plants were cultivated for 3 days in half-strength MS medium supplemented with 0 (control), 20, 40, or 60 µM CuSO_4_. Values represent the mean ± SD from three independent biological replicates. Statistically significant differences (*p* < 0.05) among experimental groups are denoted above the bars by different lowercase letters (for allantoate and allantoin) and uppercase letters (for total ureides), based on two-way ANOVA followed by Tukey’s post hoc test. For allantoate content in *aln* mutants, an independent one-way ANOVA followed by Tukey’s post hoc test was performed to specifically highlight the effect of Cu concentrations within each mutant genotype (shown in the insets). Detailed statistical outputs for the main factors (genotype and Cu concentration) and their interactions are provided in Supplementary Table S3.

Under control conditions (0 µM Cu), baseline allantoate levels in the *aln-1* and *aln-4* mutant lines were approximately 2.5-fold lower compared to WT plants (Figure 1). Conversely, allantoin content in both *aln* mutants was approximately 3-fold higher than in WT, resulting in a markedly larger total ureide pool under non-stressed conditions.

In response to copper stress, allantoate content in WT plants increased progressively with rising Cu concentrations, nearly doubling at 60 µM Cu relative to the control (Figure 1). In contrast, allantoin levels in WT remained largely unchanged across all Cu treatments, resulting in a 25% rise in total ureides at the highest Cu concentration. In the *aln-1* and *aln-4* mutants, Cu exposure induced a pronounced accumulation of allantoin, with levels increasing by approximately 30% at 60 µM Cu compared with the respective controls. Allantoate content also increased in both mutant lines in response to Cu stress, although its levels remained consistently lower than in WT plants. Consequently, total ureide content increased in a concentration-dependent manner in both *aln* lines, rising by approximately 35% at the highest Cu concentration and reaching levels more than twice those observed in WT plants under the same conditions.

Overall, Cu stress promotes ureide accumulation in all genotypes but elicits distinct metabolic responses. In WT plants, this increase is driven mainly by allantoate accumulation, whereas in the *aln* mutants, both allantoin and allantoate accumulate, resulting in a substantially larger total ureide pool. The accumulation of allantoate in both genotypes further suggests that Cu stress may also impair downstream allantoate metabolism, contributing to ureide accumulation independently of *ALN* function.

### 2.2. Impact of Cu Stress on Biomass, Lipid Peroxidation, and ROS Accumulation

**Figure 2:**
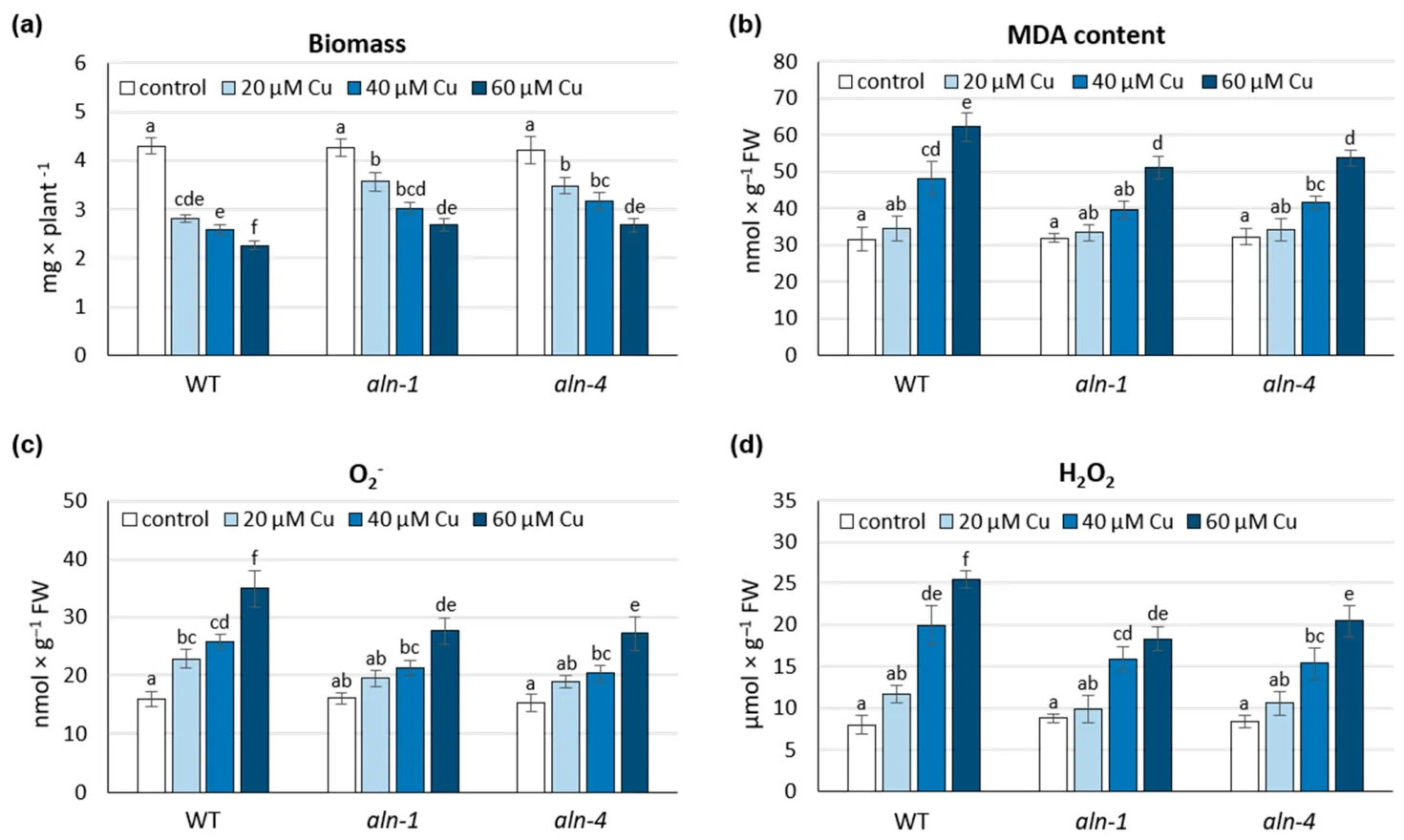
Impact of copper exposure on dry weight (**a**), malondialdehyde (MDA) content (**b**), O_2_^−^ levels (**c**), and H_2_O_2_ accumulation (**d**) in wild-type (WT) plants and mutant lines (*aln-1*, *aln-4*). Plants were cultivated for 3 days in half-strength MS medium supplemented with 0 (control), 20, 40, or 60 µM CuSO_4_. Data are presented as the mean ± SD from three independent biological replicates. Statistically significant differences (*p* < 0.05) among all experimental groups are indicated above the bars by distinct letters, based on the interaction effects of two-way ANOVA followed by Tukey’s post hoc test. Comprehensive statistical outputs for the main factors (genotype and Cu concentration) and their interactions are summarized in Supplementary Table S3.

Copper stress induced a concentration-dependent decline in biomass accumulation (dry weight, DW) and a progressive rise in malondialdehyde (MDA) content across all tested genotypes, with both effects being significantly more pronounced in WT plants than in the *aln* mutants (Figure 2a,b). In WT, biomass decreased by approximately 35% and 50% at 20 and 60 µM Cu, respectively, which coincided with a nearly 55% increase in MDA accumulation at 40 µM Cu and a two-fold rise relative to the control at 60 µM Cu. In contrast, *aln-1* and *aln-4* plants exhibited higher stress tolerance; their biomass reduction reached only around 15% at 20 µM Cu and 35% at 60 µM Cu, while lipid peroxidation was markedly attenuated, showing no significant MDA changes at 20 µM Cu and remaining about 20% lower than in WT at 60 µM Cu.

Reactive oxygen species accumulation followed a similar pattern. Copper treatment increased O_2_^−^ generation in all genotypes (Figure 2c), with WT showing the highest accumulation (~120% increase at 60 µM Cu compared to the control). In both *aln* mutants, O_2_^−^ accumulation was significantly lower, remaining approximately 22% below WT values at 60 µM Cu. Likewise, H_2_O_2_ content increased with higher Cu concentrations across all lines (Figure 2d), but the increase was substantially greater in WT plants (~220% at 60 µM Cu). In *aln-1* and *aln-4* lines, H_2_ O_2_ accumulation was lower by 20–30% compared to WT across the respective Cu treatments.

Overall, these results show that the loss of *ALN* function reduces Cu-induced lipid peroxidation and ROS accumulation, thereby supporting better maintenance of growth under copper stress.

### 2.3. Impact of Cu Stress on Antioxidant Enzyme Activities

**Figure 3:**
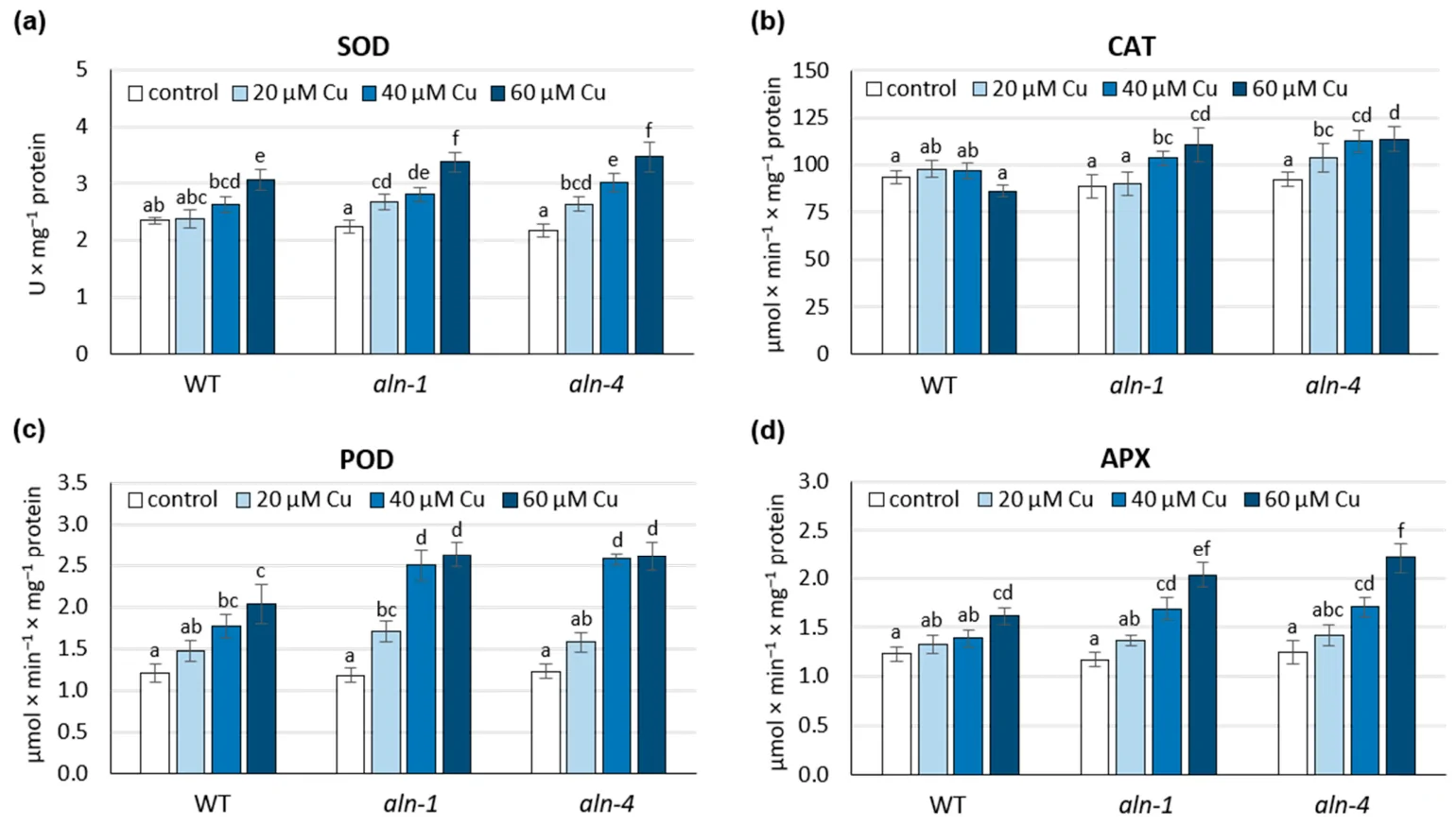
Impact of copper exposure on superoxide dismutase (SOD) (**a**), catalase (CAT) (**b**), peroxidase (POD) (**c**), and ascorbate peroxidase (APX) (**d**) activities in wild-type (WT) plants and mutant lines (*aln-1*, *aln-4*). Plants were cultivated for 3 days in half-strength MS medium supplemented with 0 (control), 20, 40, or 60 µM CuSO_4_. Data are presented as the mean ± SD from three independent biological replicates. Statistically significant differences (*p* < 0.05) among all experimental groups are indicated above the bars by distinct letters, based on the interaction effects of two-way ANOVA followed by Tukey’s post hoc test. Comprehensive statistical outputs for the main factors (genotype and Cu concentration) and their interactions are summarized in Supplementary Table S3.

Under control conditions (0 µM Cu), baseline activities of SOD, CAT, POD, and APX did not differ significantly among genotypes (Figure 3a–d).

Copper exposure modulated antioxidant responses in a dose-dependent manner, inducing statistically significant increases across all genotypes primarily at 40 and 60 µM Cu. However, this response was markedly stronger in *aln* mutants than in WT plants. SOD activity rose progressively with increasing Cu levels; however, while WT plants showed a ~30% increase at 60 µM Cu relative to the control, *aln-1* and *aln-4* mutants exhibited a stronger induction, reaching an increase of approximately 50% and nearly 60% above control values at maximum Cu concentration, respectively (Figure 3a). A distinct profile was observed for CAT activity; while no significant changes were detected in WT plants across Cu concentrations, both *aln* lines maintained elevated activity under stress, increasing by around 25% at 60 µM Cu and exceeding WT levels by approximately 30% under the same conditions (Figure 3b). POD activity was significantly enhanced by Cu exposure in all genotypes, but while WT plants displayed a moderate response, *aln-1* and *aln-4* mutants showed a more pronounced induction, exceeding a 2-fold increase at 40 and 60 µM Cu compared to their respective controls (Figure 3c). Similarly, APX activity in WT increased by ~30% at 60 µM Cu, whereas in *aln* mutants it rose almost 75% at maximum toxicity, resulting in activity levels up to 35% higher than in WT under the same stress conditions (Figure 3d).

Taken together, the enhanced enzymatic ROS-scavenging capacity of *aln* mutants under elevated Cu concentrations is directly reflected in reduced O_2_^−^, H_2_O_2_, and MDA levels (Figure 2b–d), ultimately contributing to more effective biomass retention in these lines (Figure 2a).

### 2.4. Antioxidant Gene Expression in Response to Copper Toxicity

**Figure 4:**
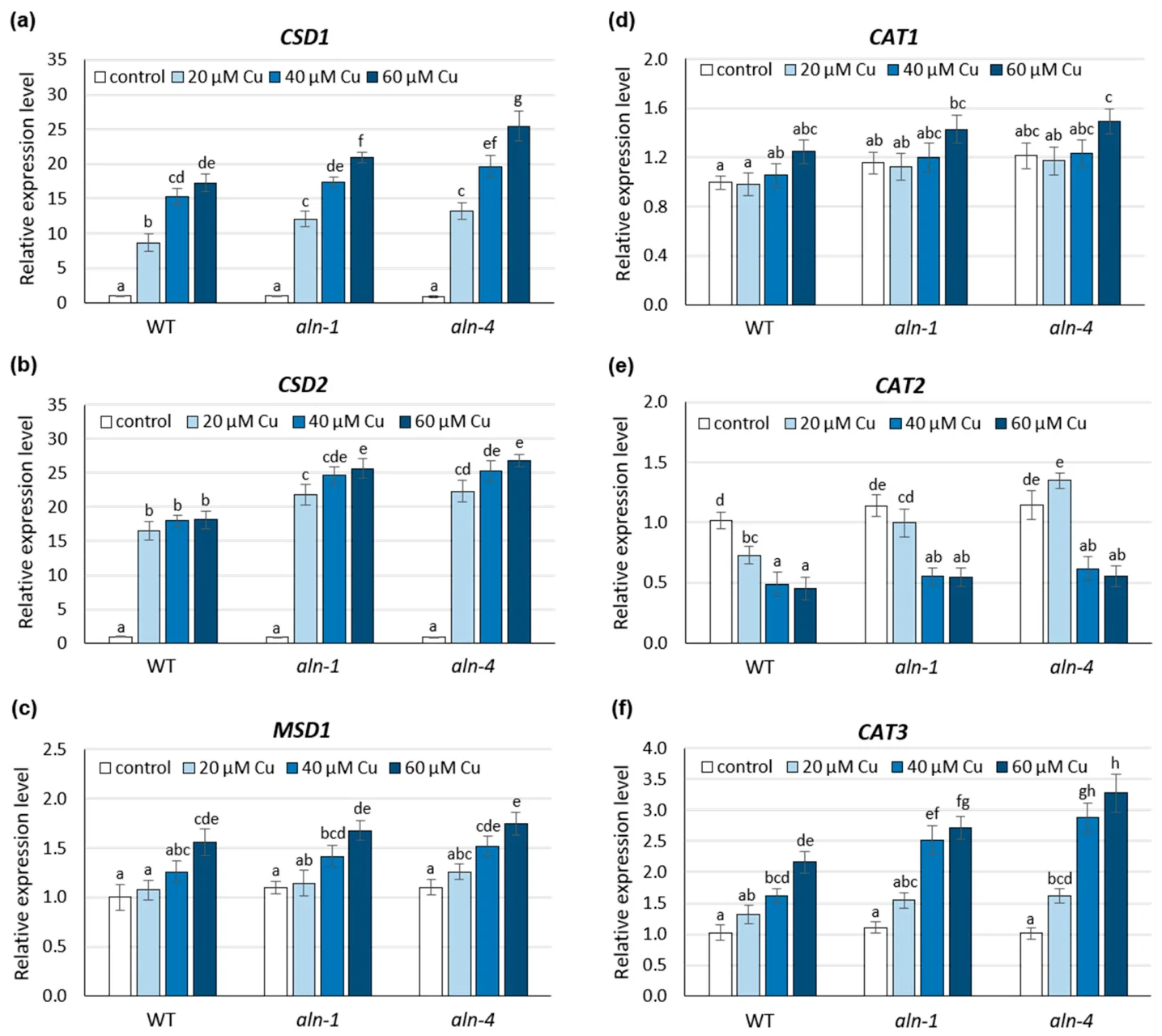
Impact of Cu stress on the relative transcript levels of *SOD* and *CAT* genes in WT and *aln* mutants (*aln-1*, *aln-4*): cytosolic Cu/Zn-SOD (*CSD1*, (**a**)), chloroplastic Cu/Zn-SOD (*CSD2*, (**b**)), mitochondrial Mn-SOD (*MSD1*, (**c**)), *CAT1* (**d**), *CAT2* (**e**), and *CAT3* (**f**). Plants were cultivated for 3 days in half-strength MS medium supplemented with 0 (control), 20, 40, or 60 µM CuSO4. Data are presented as the mean ± SD from three independent biological replicates. Statistically significant differences (*p* < 0.05) among all experimental groups are indicated above the bars by distinct letters, based on the interaction effects of two-way ANOVA followed by Tukey’s post hoc test. Comprehensive statistical outputs for the main factors (genotype and Cu concentration) and their interactions are summarized in Supplementary Table S3.

**Figure 5:**
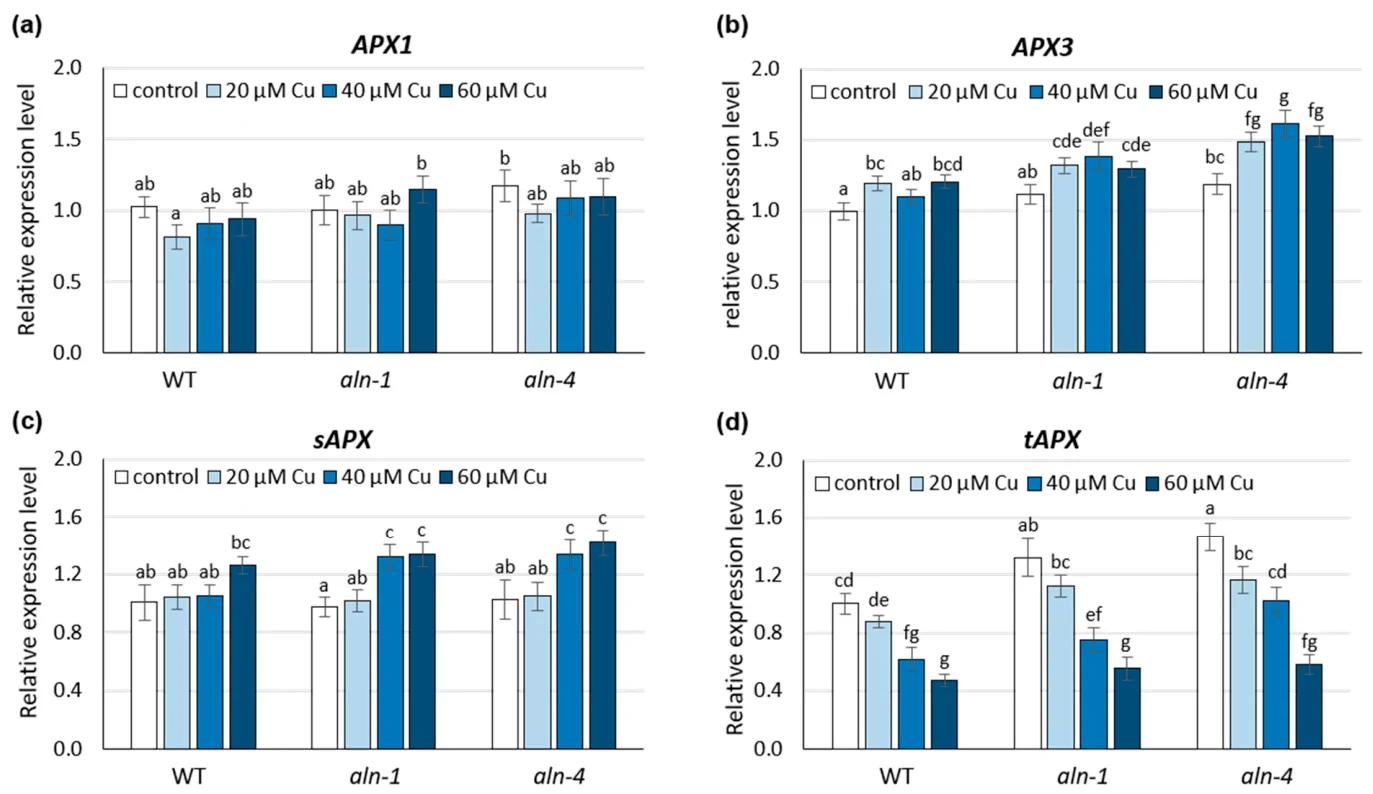
Impact of Cu stress on the relative transcript levels of genes encoding ascorbate peroxidase isoforms in WT and *aln* mutants (*aln-1*, *aln-4*): *APX1* (**a**), *APX3* (**b**), *sAPX* (**c**), and *tAPX* (**d**). Plants were cultivated for 3 days in half-strength MS medium supplemented with 0 (control), 20, 40, or 60 µM CuSO4. Data are presented as the mean ± SD from three independent biological replicates. Statistically significant differences (*p* < 0.05) among all experimental groups are indicated above the bars by distinct letters, based on the interaction effects of two-way ANOVA followed by Tukey’s post hoc test. Comprehensive statistical outputs for the main factors (genotype and Cu concentration) and their interactions are summarized in Supplementary Table S3.

**Figure 6:**
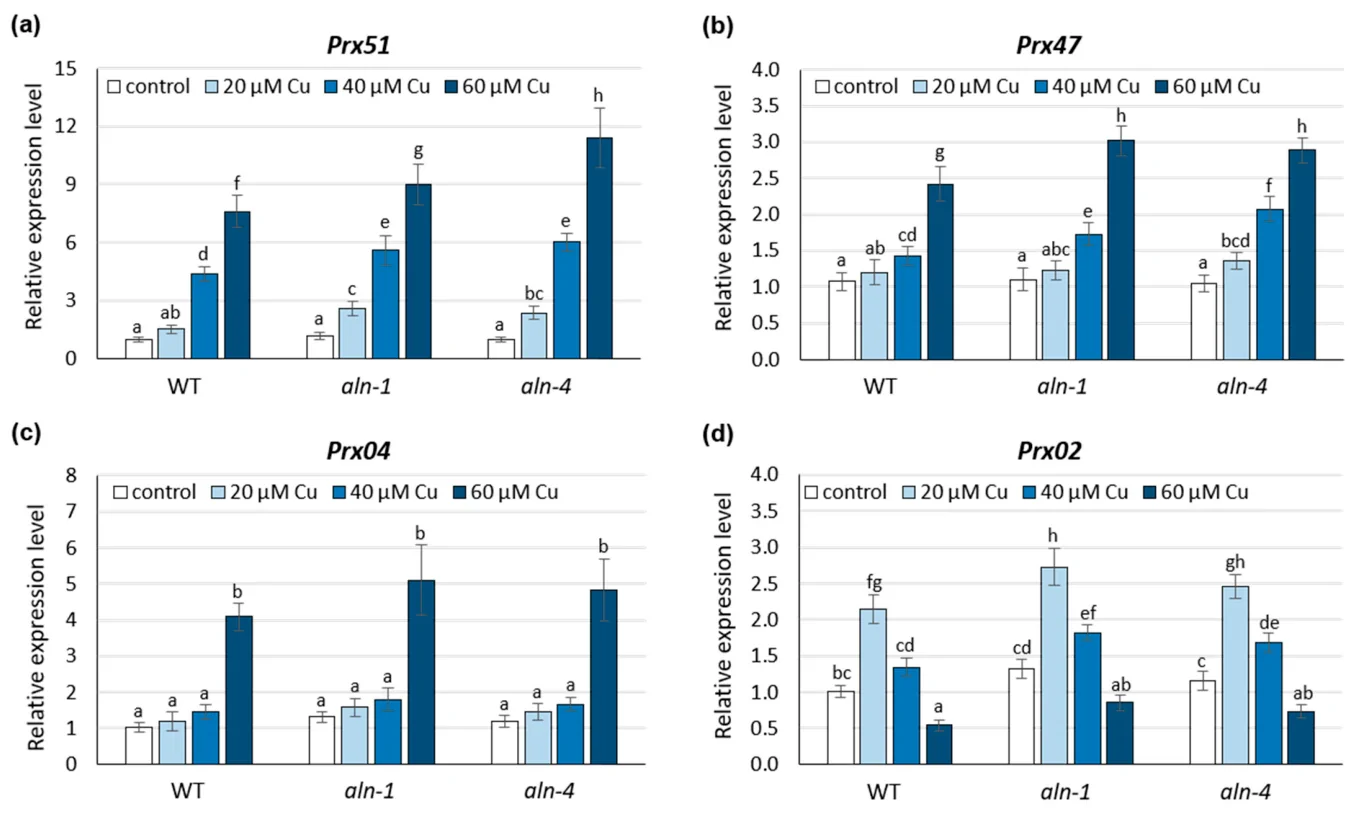
Impact of Cu stress on the relative transcript levels of genes encoding class III peroxidase isoforms in WT and *aln* mutants (*aln-1*, *aln-4*): *Prx51* (**a**), *Prx47* (**b**), *Prx04* (**c**), and *Prx02* (**d**). Plants were cultivated for 3 days in half-strength MS medium supplemented with 0 (control), 20, 40, or 60 µM CuSO4. Data are presented as the mean ± SD from three independent biological replicates. Statistically significant differences (*p* < 0.05) among all experimental groups are indicated above the bars by distinct letters, based on the interaction effects of two-way ANOVA followed by Tukey’s post hoc test. Comprehensive statistical outputs for the main factors (genotype and Cu concentration) and their interactions are summarized in Supplementary Table S3.

To investigate whether the observed enzymatic responses to excess Cu were regulated at the transcriptional level, we analyzed the relative transcript abundance of key antioxidant gene families across a range of Cu concentrations in both WT and *aln* mutant lines (Figure 4, Figure 5 and Figure 6).

Under non-stress conditions, baseline gene expression was comparable between WT plants and *aln* mutants, except for *APX3* and *tAPX*, which encode peroxisomal and thylakoid membrane-associated APX isoforms, respectively, and were higher in the mutant lines. Upon exposure to excess Cu, dynamic, isoform- and compartment-specific expression patterns emerged, with *aln-1* and *aln-4* mutants showing a significantly stronger induction of antioxidant gene expression than WT plants.

Transcript levels of *CSD1* and *CSD2* (encoding cytosolic and chloroplastic Cu/Zn-SODs, respectively) were strongly induced by Cu in a dose-dependent manner across all genotypes (Figure 4a,b). This induction was significantly higher in mutant lines; under 60 μM Cu, *CSD1* was 22% (*aln-1*) and 48% (*aln-4*) higher than in WT, while *CSD2* exceeded WT levels by 41% and 47%, respectively. Conversely, *MSD1* (encoding mitochondrial Mn-SOD) showed a modest increase, reaching levels only about 10% higher in mutants than in WT at 60 μM Cu (Figure 4c).

*CAT* gene expression was highly isoform- and genotype-dependent. While *CAT1* transcript levels showed no statistically significant differences across treatments despite a slight increase at 60 μM Cu (Figure 4d), *CAT2* expression was markedly suppressed under 40–60 μM Cu in all genotypes (Figure 4e). In contrast, *CAT3* expression was strongly upregulated by Cu treatment and showed clear genotype-specific differences, with the strongest response at 60 μM Cu, where transcript levels in *aln-1* and *aln-4* were higher than in WT by 25% and 51%, respectively (Figure 4f).

Expression analysis of genes encoding APX isoforms targeted to different subcellular compartments revealed distinct patterns in response to increasing Cu concentration. While the transcript level of cytosolic *APX1* remained largely unaltered by Cu stress in all genotypes (Figure 5a), expression of peroxisomal *APX3* increased most notably in *aln-1* and *aln-4* mutants, where at 40 μM Cu it was higher than in WT by 25% and 55%, respectively (Figure 5b). Stromal *sAPX* transcripts also accumulated in a dose-dependent manner, increasing by up to ~35% across genotypes at 60 μM Cu and maintaining higher levels in *aln* mutants than in WT at 40 μM Cu (Figure 5c). Conversely, expression of *tAPX* was progressively suppressed by Cu; nevertheless, *aln* lines consistently maintained higher expression than WT, exceeding wild-type levels by roughly 25% at 20 μM Cu (Figure 5d).

Genes encoding class III peroxidases also displayed distinct profiles. Apoplastic *Prx51* and *Prx47* were progressively induced by Cu, peaking at 60 μM Cu (Figure 6a,b). At this concentration, *Prx51* expression was 20% and 50% higher in *aln-1* and *aln-4*, respectively, compared to WT, while *Prx47* levels were 25% higher across both mutants. *Prx04* showed threshold induction exclusively at 60 μM Cu, with transcript levels approximately 20% higher in the mutants than in WT (Figure 6c). In contrast, *Prx02* peaked early at 20 μM Cu, reaching levels 27% higher in *aln-1* and 14% higher in *aln-4*, then sharply declined across all genotypes (Figure 6d).

Taken together, these findings indicate that the enhanced copper tolerance observed in *aln-1* and *aln-4* is associated with substantial transcriptional reprogramming of antioxidant defense pathways. The preferential and higher transcript abundance of specific antioxidant isoforms—most notably *CSD1*, *CSD2*, *CAT3*, *APX3*, *tAPX*, *Prx51*, and *Prx47* in *aln* mutants —may contribute to a more effective ROS detoxification mechanism under copper toxicity.

## 3. Discussion

Allantoin accumulation has been associated with plant responses to various abiotic stresses [12], yet the effects of copper (Cu) stress on ureide accumulation and changes in the ureide profile remain unknown. In WT *Arabidopsis thaliana*, allantoin was the predominant ureide under control conditions, whereas increasing Cu concentrations progressively shifted the ureide profile toward allantoate, which became predominant at 60 µM Cu (Figure 1). This shift resulted primarily from increased allantoate levels, while allantoin remained relatively stable, indicating that Cu stress alters ureide partitioning rather than substantially increasing the total ureide pool. In contrast, the *aln-1* and *aln-4* loss-of-function mutants displayed a markedly altered ureide profile compared with WT plants, consistent with impaired *ALN*-mediated conversion of allantoin to allantoate (Figure 1). Under Cu stress, both ureides accumulated in the *aln* mutants, but *ALN* deficiency favored allantoin retention, resulting in a greater expansion of the total ureide pool. The increase in allantoate levels despite *ALN* deficiency further suggests that Cu-induced changes in ureide metabolism may extend beyond the ALN-catalyzed step and involve downstream processes that affect allantoate turnover.

The altered ureide profile of the *aln* mutants was associated with enhanced tolerance to Cu stress. Compared with WT plants, *aln-1* and *aln-4* maintained higher biomass and exhibited lower levels of MDA, superoxide anions, and hydrogen peroxide under Cu exposure, indicating reduced oxidative damage and improved ROS homeostasis (Figure 2). This is consistent with previous studies linking allantoin accumulation—whether resulting from the loss of *ALN* function or exogenous application—with increased tolerance to cadmium [22,36], salt [23,24], drought, and osmotic stress [35], which was likewise associated with reduced ROS accumulation and lipid peroxidation. Our findings extend these observations to Cu toxicity and suggest that the shift in the ureide profile toward allantoin observed in the *aln* mutants may contribute to improved protection against Cu-induced oxidative stress, although the specific contribution of allantoin itself remains to be established.

The enhanced Cu tolerance of the *aln* mutants was accompanied by a stronger activation of the antioxidant enzyme system. Cu exposure induced SOD, CAT, POD, and APX activities more strongly in the *aln* mutants than in WT plants (Figure 3), indicating a coordinated enhancement of enzymatic ROS detoxification. As shown previously, the responses of individual antioxidant enzymes to Cu stress vary considerably among plant species, genotypes, Cu concentrations, and exposure durations. In Arabidopsis, Cu excess was reported to increase SOD and peroxidase activities while decreasing CAT activity [37,38], a pattern also observed in maize, where Cu increased SOD, POD, and APX but reduced CAT activity [39]. In contrast, short-term Cu exposure in maize induced SOD, CAT, and APX activities, indicating that coordinated activation of these enzymes can also constitute an early response to Cu-induced oxidative stress [40]. Moreover, differences in the strength of antioxidant responses between Cu-tolerant and Cu-sensitive maize genotypes suggest that more robust, coordinated activation of antioxidant enzymes may contribute to Cu tolerance [41]. In our study, the more Cu-tolerant *aln* mutants exhibited a stronger antioxidant response, with Cu stress inducing SOD, POD, and APX activities more markedly than in WT plants, while CAT activity was significantly increased only in the mutants. The combined enhancement of enzymes responsible for superoxide dismutation and H_2_O_2_ detoxification likely increased the efficiency of ROS scavenging, thereby limiting oxidative damage, as reflected by the lower ROS and MDA levels observed in the mutants (Figure 2b–d). These results suggest that enhanced antioxidant capacity represents an important component of the improved Cu tolerance associated with *ALN* deficiency.

To gain further insight into the molecular basis of this enhanced antioxidant capacity, we analyzed the expression of genes encoding major antioxidant enzymes. The transcriptional data revealed a broad reorganization of antioxidant defense in the *aln* mutants. Changes in antioxidant gene expression complemented the stronger Cu-induced activities of SOD, CAT, POD, and APX, indicating that *ALN* deficiency enhances antioxidant protection at both transcriptional and enzymatic levels. Rather than a uniform activation of antioxidant genes, the response involved selective regulation of individual isoforms, consistent with the complex, isoform-specific, and compartment-dependent regulation of antioxidant systems under heavy metal stress [38].

Increased expression of *CSD1* and *CSD2* suggests enhanced capacity to control superoxide radicals in the cytosol and chloroplasts (Figure 4a–c), respectively, as these genes encode cytosolic and chloroplastic Cu/Zn-superoxide dismutase isoforms [42,43]. The higher expression of *CSD2* may be particularly relevant under Cu stress, as excess Cu can impair chloroplast function and photosynthetic electron transport, potentially promoting ROS accumulation [44,45]. Previous studies have demonstrated that Cu and Cd induce distinct transcriptional responses of *SOD* genes. In Arabidopsis, Cu excess stimulated the expression of *CSD1* and *CSD2*, whereas *FSD1* was repressed, while Cd stress induced *FSD1* and reduced *CSD2* transcript abundance, highlighting the metal-specific regulation of SOD isoforms [46]. The simultaneous induction of *CSD1* and *CSD2* observed in the *aln* mutants is therefore consistent with enhanced superoxide detoxification capacity in both cytosolic and chloroplastic compartments during Cu stress. Interestingly, *CSD2* was also preferentially induced in *TsPAP1*-overexpressing plants under Cu stress [38]. This suggests that strengthening chloroplastic ROS protection may represent a common component of Cu tolerance, while *ALN* deficiency may provide additional protection by reinforcing cytosolic superoxide scavenging.

The isoform-specific regulation of catalase genes further highlights the complex antioxidant adaptation associated with *ALN* deficiency. The *Arabidopsis* catalase family comprises three members—*CAT1*, *CAT2*, and *CAT3*—which differ in their expression patterns and physiological roles [47]. Among them, *CAT1* is primarily involved in ROS regulation during seed germination and is strongly induced by hormonal and environmental stresses, such as drought, salinity, and heavy metal exposure [38,46,47,48,49]. In line with its stress-responsive nature, *CAT1* transcript levels in our study were markedly induced; however, only under severe toxicity (60 µM Cu) (Figure 4d). In contrast, *CAT2* primarily scavenges photorespiratory H_2_O_2_ to maintain photosynthetic redox balance, whereas *CAT3* participates in development, senescence, and specific oxidative responses [47,50,51], with double-mutant analyses supporting their overlapping roles in leaves [52]. In our experiments, Cu stress suppressed *CAT2* expression (Figure 4e), consistent with earlier findings [38], although *aln* mutants retained higher *CAT2* transcript levels compared to the WT, especially at 20 µM Cu. Conversely, Cu upregulated *CAT3* expression across all genotypes, with a significantly stronger response in *aln* mutants (Figure 4f). Collectively, these findings demonstrate that *ALN* deficiency triggers a distinct, isoform-specific modulation of individual *CAT* genes under Cu stress rather than a uniform activation of the catalase network.

A similar isoform-dependent regulation was observed for ascorbate peroxidases (APXs), which provide compartment-specific control of H_2_O_2_ metabolism. Unlike catalases, which act primarily within peroxisomes to drive bulk H_2_O_2_ removal, APX isoforms are distributed across multiple cellular compartments, enabling localized ROS regulation. Cytosolic APX1 modulates cytoplasmic oxidative signaling, stromal (sAPX) and thylakoid (tAPX) isoforms protect chloroplasts during photosynthetic electron transport, and APX3 controls peroxisomal H_2_O_2_ [53,54]. Heavy metal stress triggers complex, isoform-specific shifts in APX expression that reflect local ROS-generating sites and signaling networks, such as OXI1-dependent cascades under Cu excess [46]. In the present study, *aln* mutants exhibited higher transcript levels of *APX3*, *sAPX*, and *tAPX* under Cu stress compared with WT plants (Figure 5a-d). Up-regulation of chloroplastic *APXs* is particularly vital to mitigate Cu-induced damage to the photosynthetic apparatus [55], while elevated *APX3* transcripts reinforce peroxisomal defenses [56]. Together, the coordinated modulation of CAT and APX isoforms indicates that altered ureide metabolism in *aln* mutants drives a multi-compartmental reorganization of antioxidant capacity rather than triggering a single ROS-scavenging pathway.

A similar, highly specific pattern was observed for class III peroxidases, further supporting the concept of a strategically reorganized antioxidant response in *aln* mutants. Class III peroxidases comprise a large, functionally diverse gene family involved in extracellular ROS detoxification, stress signaling, and cell wall remodeling, including lignification [57,58]. Individual *Arabidopsis Prx* genes display distinct spatial and developmental expression profiles, reflecting high functional specialization within the apoplast [59]. In the present study, Cu stress induced several key *Prx* genes—including *Prx04*, *Prx47*, and *Prx51*—confirming their role in the basal plant response to Cu toxicity (Figure 6a–d). However, their consistently higher transcript accumulation in *aln* mutants suggests that altered ureide metabolism amplifies this apoplastic defense. Specifically, enhanced expression of *Prx04* and *Prx47* likely supports cell wall reinforcement and redox homeostasis; as *Prx04* has previously been associated with lignification [60], while *Prx47* is linked to vascular development and oxidative wall metabolism [61]. The robust upregulation of *Prx51* in *aln* mutants is particularly intriguing, as it highlights targeted isoform modulation rather than generalized family activation. Notably, *Prx51* was also upregulated under Cu stress in *TsPAP1*-overexpressing lines [38]. Its co-induction across two genetically distinct, Cu-tolerant *Arabidopsis* systems suggests that *Prx51* represents a conserved component of apoplastic ROS regulation under metal excess, though its precise mechanical contribution to Cu tolerance warrants further.

Together, these transcriptional findings indicate that *ALN* deficiency promotes a selective, compartment-specific remodeling of antioxidant defenses rather than a uniform upregulation of ROS-scavenging genes. This targeted regulation of *CSD*, *CAT*, *APX*, and *Prx* gene expression closely aligns with the elevated enzymatic activities of SOD, CAT, POD, and APX in *aln* mutants, ultimately leading to lower superoxide and H_2_O_2_ accumulation, reduced lipid peroxidation, and preserved growth under Cu excess. Notably, a comparison with *TsPAP1*-overexpressing lines reveals that distinct metabolic pathways conferring Cu tolerance recruit overlapping yet non-identical antioxidant components [38]. Ultimately, these results indicate that rather than acting as a direct ROS scavenger, altered ureide partitioning functions as a metabolic trigger that primes multi-compartmental defenses to enhance Cu tolerance in *Arabidopsis*.

## 4. Materials and Methods

### 4.1. Plant Material and Experimental Conditions

Two T-DNA insertion mutant lines of the *ALN* gene (At4g04955) and their corresponding wild-type *Arabidopsis thaliana* ecotype Columbia-0 (Col-0) were used in this study. The mutant lines SALK_000325 (NASC ID: N500325) and SALK_013427 (NASC ID: N661546), hereafter referred to as *aln-1* and *aln-4*, respectively, were generated at the Salk Institute for Biological Studies (La Jolla, CA, USA), and seeds were obtained from the Nottingham Arabidopsis Stock Center (NASC, Nottingham, UK). The *aln-1* line was previously used and described by Watanabe et al. [35] and Takagi et al. [62], whereas *aln-4* was characterized by Todd and Polacco [63] and Soltabayeva et al. [16].

To obtain the homozygous mutant lines, seeds were sown in a mixture of commercial potting soil, perlite, and sand (3:1:1, *v*/*v*/*v*). Following the development of true leaves, seedlings were transplanted into individual pots and cultivated in a growth chamber under controlled conditions (200 μE × m^−2^ × s^−1^), 22 °C during a 14 h light period and 19 °C during a 10 h dark period. Young leaves were harvested from the developed rosettes for genomic DNA extraction and subsequent genotyping to identify homozygous mutant plants.

To study the effects of copper on the antioxidant defense system, plant material was grown in an axenic flask culture system, following a modified protocol adapted from Hétu et al. [64] and Zhao et al. [65]. Seeds collected from homozygous *Arabidopsis thaliana* mutant and wild-type (WT) plants were surface-sterilized by immersion in 96% ethanol for 30 s, rinsed with sterile distilled water, and subsequently treated with 10% sodium hypochlorite for 3 min [66]. After three washes with sterile distilled water, seeds were sown onto Petri dishes containing solid ½ MS medium (0.8% agar). Plates were first incubated at 4 °C for 2 days in the dark to synchronize seed germination, then transferred to a growth chamber and incubated under the same conditions as described above. After 6 days, uniformly developed seedlings were transferred to fresh Petri dishes with solid ½ MS medium (0.55% agar). Ten seedlings per plate were placed onto sterile stainless steel mesh pieces (4 × 7 cm; wire diameter: 0.16 mm, mesh aperture: 0.4 mm) layered on the surface of the medium, and the plates were positioned vertically for another 6 days of seedling growth. Afterward, the seedlings were thinned to eight uniform individuals per mesh. The short ends of each mesh were then bent downward by approximately 3 mm to form a raised platform. The seedlings, together with their supporting mesh, were transferred into 250 mL wide-mouth Erlenmeyer flasks containing 10 mL of sterile liquid ½ MS medium, keeping the shoots elevated above the liquid. The plants were then cultivated under gentle agitation for an additional 6 days in basal ½ MS medium. The medium was replaced every three days. Then, the ½ MS medium was supplemented with 0 (control) 20, 40, or 60 μM CuSO_4_. Copper concentrations were selected based on the literature [67] and preliminary dose–response assays to represent an oxidative stress gradient: 20 μM represents a mild stress level that inhibits plant growth without inducing oxidative damage measurable by our analytical methods (Section 4.4); 40 μM is the threshold concentration that triggers statistically significant oxidative stress; and 60 μM represents severe toxicity. After 72 h of treatment, plants were harvested, rinsed with distilled water, blotted dry, frozen in liquid nitrogen, and stored at −80 °C until further analysis. The experiment was performed in three independent biological replicates.

### 4.2. Genotyping of aln Mutants

Genomic DNA was isolated using the cetyltrimethylammonium bromide (CTAB) extraction method [68]. To identify homozygous T-DNA insertion lines, PCR-based genotyping was performed using the isolated genomic DNA as a template. The reaction set included the left border T-DNA primer (LBb1.3) and *ALN* gene-specific primers flanking the insertion site on the left (L) or right (R) side, designed via the Salk Institute Genomic Analysis Library (SIGnAL) tool (http://signal.salk.edu/tdnaprimers.2.html; accessed on 11 February 2023). Primers aln-1L and aln-1R were used for genotyping *aln-1* mutants, whereas primers aln-4L and aln-4R were used for *aln-4* plants (Table S1). The genotyping results are shown in Figures S2 and S3.

### 4.3. Quantification of Ureides

To quantify ureide content, plant samples were homogenized in a 1:3 (*w*/*v*) ratio with 50 mM potassium phosphate buffer (pH 7.0), heat-treated at 100 °C for 20 min, and centrifuged twice (16,000× *g*, 20 min, 4 °C) [22]. The concentrations of allantoin and allantoate in the obtained supernatants were determined through differential conversion of ureides into glyoxylate, followed by colorimetric assay of glyoxylate derivatives [69,70].

### 4.4. Determination of Malondialdehyde, O_2_^−^ and H_2_O_2_ Content

Lipid peroxidation was evaluated by measuring malondialdehyde (MDA) content according to Heath and Packer [71], with minor modifications [22]. Fresh plant material (150 mg) was ground in 1.5 mL of 0.1 M potassium phosphate buffer (pH 6.8). The resulting homogenates were centrifuged at 13,000× *g* for 15 min. Supernatants were collected, mixed in a 1:1 ratio with a TBA reagent (0.5% thiobarbituric acid in 15% trichloroacetic acid), and heated at 100 °C for 20 min. Following rapid cooling, the mixtures were centrifuged again (13,000× *g*, 10 min), and the absorbance of the supernatant was recorded at 532 nm and 600 nm. MDA concentrations were determined using a standard curve.

Superoxide anion (O_2_^−^) content was determined based on the method of Elstner and Heupel [72], as modified by [22]. Plant tissues were ground (1:4, *w*/*v*) in 65 mM potassium phosphate buffer (pH 7.8) supplemented with 7.5 mM hydroxylamine hydrochloride. The homogenates were centrifuged at 13,000× *g* (4 °C, 15 min), and a 0.1 mL aliquot of the supernatant was combined with 0.17 mL of extraction buffer and 0.03 mL of 50 mM hydroxylamine hydrochloride. After incubating at 25 °C for 20 min, 0.3 mL of 58 mM sulfanilamide and 0.3 mL of 7 mM N-(1-naphthyl)ethylenediamine dihydrochloride were added, followed by an additional 20 min incubation at 25 °C. The reaction mixture was then extracted with chloroform and centrifuged (13,000× *g*, 5 min). The absorbance of the upper aqueous layer was read at 530 nm, and O_2_^−^ concentrations were calculated against a sodium nitrite (NaNO_2_) standard calibration curve.

Determination of H_2_O_2_content was carried out according to Jana and Choudhuri [73] with minor adaptations. Plant material was ground in 50 mM phosphate buffer (pH 6.5, 1:10 ratio) and centrifuged for 25 min at 6000× *g*. After mixing 0.6 mL of extract with 0.2 mL titanium reagent (0.1% TiCl_4_ in 20% H_2_SO_4_) and re-centrifuging (6000 × *g*, 15 min), optical density was measured at 410 nm. Final values were calculated using a standard curve.

### 4.5. Determination of Antioxidant Enzyme Activities

Soluble plant proteins were extracted in an ice-cold buffer (1:5, *w*/*v* ratio; 0.1 g tissue per 0.5 mL buffer) containing 50 mM potassium phosphate (pH 7.0), 1 mM EDTA, 1% (*w*/*v*) PVP-40, 1 mM PMSF, 2 mM *β*-mercaptoethanol, and 1 mM ascorbic acid. The homogenates were centrifuged at 16,000× *g* (4 °C, 20 min), and the resulting supernatants were used for assaying antioxidant enzyme activities according to established procedures. Superoxide dismutase (SOD) activity was assayed by measuring its ability to inhibit the photoreduction of p-nitroblue tetrazolium (NBT), as described by Giannopolitis and Ries [74]. The 3 mL reaction mixture comprised 50 mM potassium phosphate buffer (pH 7.8), 13 µM methionine, 75 µM NBT, 100 µM EDTA, 2 µM riboflavin, and 50 µL of enzyme extract. Samples were illuminated for 30 min using 40 W fluorescent lights at a distance of 10 cm. One unit of SOD activity was defined as the amount of enzyme required to produce a 50% inhibition of NBT photoreduction at 560 nm. Catalase (CAT) activity was measured by monitoring the decrease in absorbance at 240 nm resulting from H_2_O_2_ decomposition [75]. The assay mixture (2.0 mL total volume) consisted of 1.98 mL of 50 mM potassium phosphate buffer (pH 7.0), 1 mL of 30 mM H_2_O_2_, and 20 µL of enzyme extract. Ascorbate peroxidase (APX) activity was evaluated following Nakano and Asada [76] by monitoring the reduction in absorbance at 290 nm caused by ascorbate oxidation. The reaction mixture contained 2.91 mL of 50 mM potassium phosphate buffer (pH 7.0), 30 µL of 50 mM ascorbic acid, 30 µL of 50 mM H_2_O_2_, and 30 µL of extract. Class III peroxidase (POD) activity was determined using a modified protocol of Maehly and Chance [77], based on the H_2_O_2_-dependent oxidation of guaiacol to tetraguaiacol. The reaction mixture contained 90 µL of 50 mM sodium acetate buffer (pH 5.6), 50 µL of 20 mM guaiacol, 50 µL of 34.3 mM H_2_O_2_ and 10 µL of a 100-fold diluted enzyme extract. Enzyme activity was calculated using an extinction coefficient of 26.6 mM^−1^ cm^−1^ at 470 nm for tetraguaiacol.

### 4.6. Determination of Soluble Protein Content

Total soluble protein concentration was measured using the Bradford assay [78], with bovine serum albumin (BSA) as the standard.

### 4.7. RNA Extraction and Gene Expression Analysis

Total RNA was extracted as previously described [79] and treated with DNase I to remove genomic DNA contamination. Subsequently, 4 μg of total RNA was reverse transcribed into cDNA using the Transcriptor First Strand cDNA Synthesis Kit (Roche Diagnostics, Mannheim, Germany) in accordance with the manufacturer’s instructions. Quantitative real-time PCR was performed on a LightCycler^®^ 96 system (Roche Diagnostics, Mannheim, Germany) to evaluate target gene expression. Reactions were carried out in a final volume of 10 μL containing 5 μL of SYBR Green I Master Mix (Roche Diagnostics, Mannheim, Germany), 3 μL of cDNA template (corresponding to 10 ng of total RNA input), and 0.5 μM of each specific primer. The thermal cycling parameters were as described previously [80]. Relative transcript abundance was calculated using the 2^−ΔΔCt^ method [81]. The gene coding for glyceraldehyde 3-phosphate dehydrogenase (GAPDH) gene served as an internal reference for normalization. Specific sequences for all real-time PCR primers are provided in Supplementary Table S2 [38,82]. All assays were conducted with three technical replicates across at least three independent biological replicates.

### 4.8. Statistical Analyses

Statistical analysis was performed using data from three independent biological replicates, each with three technical replicates, and results are presented as mean ± SD. Data were analyzed using a two-way analysis of variance (ANOVA), with genotype and Cu concentration as the two main factors. Normality of the data distribution was assessed using the Shapiro–Wilk test, and homogeneity of variance was confirmed using Levene’s test. Significant differences among group means were assessed using Tukey’s post hoc test. All statistical calculations were performed using Statistica version 9.1.

## Data Availability

The original contributions presented in this study are included in the article/supplementary material. Further inquiries can be directed to the corresponding authors.

## Supplementary Materials

The following supporting information can be downloaded at: https://www.mdpi.com/article/10.3390/plants15193074/s1, Figure S1. PCR-based genotyping of *aln-1* mutants; Figure S2. PCR-based genotyping of *aln-4* mutants; Table S1. Primer sequences used for genotyping of T-DNA insertion mutant lines of the *ALN* gene (locus At4g04955); Table S2. Primer sequences used for real-time PCR analysis of the expression of *Arabidopsis thaliana* genes; Table S3. Two-way ANOVA summary of the effects of copper (Cu) concentration, genotype, and their interaction on plant traits.
