# Supplementary material for: Loss of ALN Function in Arabidopsis thaliana Induces Allantoin Accumulation and Enhances Antioxidant Enzyme Responses to Copper Toxicity
Source: Plants (Basel). 2026 Oct 8;15(19):3074. doi: 10.3390/plants15193074 (PMC13667067; doi:10.3390/plants15193074)
Supplement: Supplementary file 1 [file plants-15-03074-s001.zip › plants-4569005-supplementary.pdf]

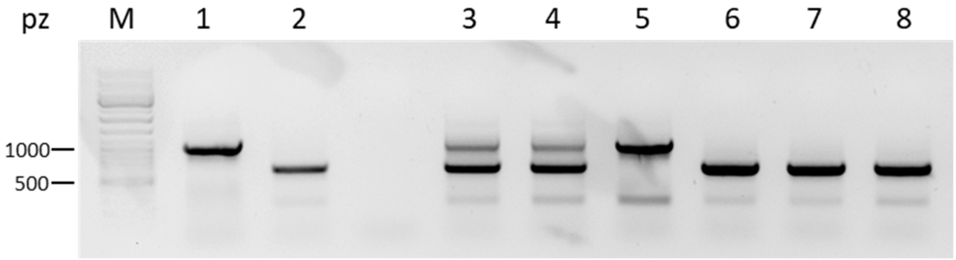

Figure S1. PCR-based genotyping of *aln-1* mutants. Representative results of agarose gel electrophoresis of PCR products are shown. M, GeneRuler DNA Ladder Mix (Thermo Fisher Scientific). Lanes 1 and 5, WT plants; lanes 2 and 6–8, homozygous mutant plants; lanes 3 and 4, heterozygous mutant plants.

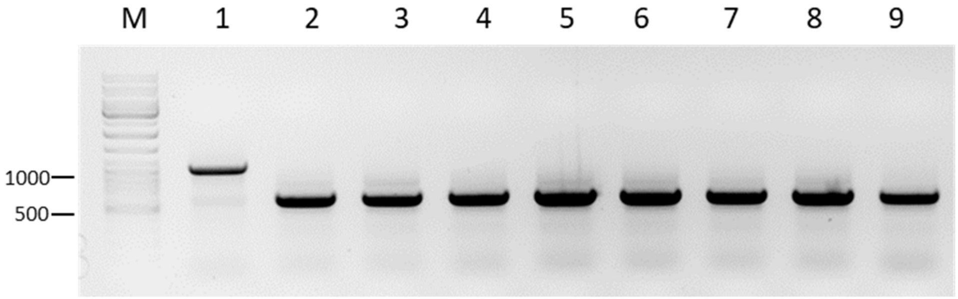

Figure S2. PCR-based genotyping of *aln-4* mutants. Representative results of agarose gel electrophoresis of PCR products are shown. M, GeneRuler DNA Ladder Mix (Thermo Fisher Scientific). Lane 1, WT plant; lanes 2–9, homozygous mutant plants.

Table S1. Primer sequences used for genotyping of T-DNA insertion mutant lines of the *ALN* gene (locus At4g04955).

Mutant name	Primer name	Oligonucleotide sequence (5'->3')	Amplicon length in WT plants (bp)	Amplicon length in homozygous *aln* lines (bp)
aln-1	aln-1L	CACGCAGAGATCGAGAGAGAC	1122	594-894
	aln-1R	TACATCAAATTCGGCTTCAGG		
	LBb1.3	ATTTTGCCGATTTCGGAAC		
aln-4	aln-4L	TGATTTTGATAAACTGATGCAAATAC	1127	546-846
	aln-4R	GCTGAGCTCTGATCATTCACC		
	LBb1.3	ATTTTGCCGATTTCGGAAC		

Table S2. Primer sequences used for real-time PCR analysis of the expression of *Arabidopsis thaliana* genes. F, forward oligonucleotide; R, reverse oligonucleotide. Gene nomenclature for *AtPrx* genes follows the PeroxiBase (formerly RedOxiBase) classification system [79]. Primer sequences were used as reported by Piątkowska et al. [38].

Gene name and	Gene locus	Oligonucleotide sequence (5'->3')	Amplicon length (bp)
CSD1 (Cu/Zn SOD)	At1g08830	F: CATGCTGGTGATCTAGGAAACA<br>R: CAACAGCCCTACCAACAATAGA	115
CSD2 (Cu/Zn SOD)	At2g28190	F: CTCCTTCTCGTCTTCTCATTCC<br>R: ATTGGAGACGGTGGAGATTG	102
MSD1 (MnSOD)	At3g10920	F: GAGAGATCATGCAGATTCATCACC<br>R: TCTCCCTTGTTCACAGCTTGATC	80
CAT1	At1g20630	F: TGCTCTGGAAATCGTGAGAAG<br>R: ATCGTTCTTGCCTGTCTGAAT	100
CAT2	At4g35090	F: CAAACCCGTGTCTTCTCCTATGC<br>R: GTTAACCTCCTCGTCCCTGTGC	152
CAT3	At1g20620	F: CTCCGGTTATTGTCCGTTTCT<br>R: CAAAGTTTCCCTCTCTGGTGT	111
AtPrx02/ AtP11	At1g05250	F: CCCTTGCCGCTAAACTTCTAA<br>R: CGTCTCTTTCCGCATCATTCT	105
AtPrx51/ AtP37	At4g37530	F: GTTTCTTGCGCTGATATTCTCACG<br>R: GCAAGATTAACGACGTCACGAG	53
AtPrx04/ AtP46	At1g14540	F: CTCTCAGGAGCTCACACATTAG<br>R: GCTTACGGGTACTGGAGAATC	106
AtPrx47/ ATP32	At4g33420	F: ATGACTTACTACATGATGAGCTGTCC<br>R: CAGTGTTGTCTTTCGTTGAATCTAG	178

APX1	At1g07890	F: CCAACCGTGAGCGAAGATTA<br>R: TCGAAAGTTCCAGCAGAGTG	128
sAPX	At4g08390	F: AGAATGGGATTAGATGACAAGGAC<br>R: TCCTTCTTTCGTGTACTTCGT	117
tAPX	At1g77490	F: GCTAGTGCCACAGCAATAGAGGAG<br>R: TGATCAGCTGGTGAAGGAGGTC	137
APX3	At4g35000	F: GGATGCTGGAACCTATGATGCTC<br>R: CAAGCTGATACAGGTCTGCGTATG	173
GAPDH	At1g13440	F: TTGGTGACAACAGGTCAAGCA<br>R: AAACTTGTCGCTCAATGCAATC	62

Table S3. Two-way ANOVA summary of the effects of copper (Cu) concentration, genotype, and their interaction on plant traits. Values displayed in the table represent the empirical Fisher's test statistics (*F*) and corresponding degrees of freedom (*df*) from the two-way ANOVA. Data are derived from *n* = 4 biological replicates. Asterisks indicate statistical significance levels: ns = non-significant ($p > 0.05$), * = $p \leq 0.05$, ** = $p \leq 0.01$, *** = $p \leq 0.001$.

**Parameter**	**Cu Concentration (*F*, *df* = 3)**	**Genotype (*F*, *df* = 2)**	**Interaction (*F*, *df* = 6)**
Dry Weight	261.850 ***	32.550 ***	6.140 ***
MDA content	123.898 ***	9.711 ***	3.083 *
O_2^- content	111.450 ***	38.310 ***	4.140 **
H_2O_2 content	145.741 ***	14.854 ***	4.502 **
Allantoin content	13.742 ***	532.386 ***	6.289 ***
Allantoic acid content	34.794 ***	100.812 ***	3.093 *
Total ureide content	33.195 ***	313.820 ***	3.064 *
SOD activity	139.738 ***	14.491 ***	4.717 ***
CAT activity	10.464 ***	14.334 ***	6.288 ***
POD activity	152.966 ***	29.327 ***	6.800 ***
APX activity	88.415 ***	19.877 ***	5.974 ***
CAT1 expression	21.210 ***	18.450 ***	1.629 ns
CAT2 expression	67.221 ***	22.083 ***	2.593 *
CAT3 expression	200.706 ***	54.194 ***	3.824 **
CSD1 expression	553.047 ***	42.687 ***	7.195 ***
CSD2 expression	913.985 ***	95.702 ***	11.948 ***
MSD1 expression	55.397 ***	8.930 ***	0.340 ns
Prx02 expression	258.716 ***	15.316 ***	1.128 ns
Prx51 expression	489.318 ***	12.873 ***	4.453 **
Prx04 expression	132.503 ***	7.616 **	1.217 ns
Prx47 expression	163.220 ***	12.721 ***	1.873 ns
APX1 expression	4.559 **	7.626 **	1.315 ns
APX3 expression	51.680 ***	247.150 ***	5.080 ***
sAPX expression	28.309 ***	5.190 *	2.273 ns
tAPX expression	129.529 ***	44.889 ***	3.475 **
